# “Staying at home” to tackle COVID-19 pandemic: rhetoric or reality? Cross-cutting analysis of nine population groups vulnerable to homelessness in Japan

**DOI:** 10.1186/s41182-020-00281-0

**Published:** 2020-11-23

**Authors:** Masami Fujita, Sadatoshi Matsuoka, Hiroyuki Kiyohara, Yousuke Kumakura, Yuko Takeda, Norimichi Goishi, Masayoshi Tarui, Masaki Inaba, Mari Nagai, Masahiko Hachiya, Noriko Fujita

**Affiliations:** 1 Bureau of International Health Cooperation, National Centre for Global Health and Medicine, 1-21-1, Toyama, Shinjuku-ku, Tokyo, 162-8655 Japan; 2 Department of Mental Health, The University of Tokyo, 7-3-1, Hongo, Bunkyo-ku, Tokyo, 113-0033 Japan; 3 Department of Medical Education, Juntendo University, 101 Amano Building, 2-8-3 Hongo, Bunkyo-ku, Tokyo, 113-0033 Japan; 4 Graduate School of Urban Management, Osaka City University, 3-3-138 Sugimoto, Sumiyoshi-ku, Osaka-shi, Osaka, Japan; 5 Faculty of Letters, Keio University, 2-15-45 Mita, Minato-ku, Tokyo, 108-8345 Japan; 6 Japan Civil Society Network on SDGs, 604 Sankyo Building Honkan, 1-7-10, Iidabashi, Chiyoda-ku, Tokyo, 102-0072 Japan

**Keywords:** Homelessness, Vulnerable populations, COVID-19, Staying at home

## Abstract

Although the “stay-at-home” order is advocated against the coronavirus disease 2019 (COVID-19), the lives of individuals lacking adequate housing are threatened. We developed a framework to assess various populations with unstable housing in terms of socio-economic consequences of COVID-19, risk of COVID-19 infection and progression, existing/urgent measures, and remaining challenges. Within the framework, nine groups vulnerable to homelessness in Japan were classified into (i) “people without accommodation,” (ii) “people living in temporary or crisis accommodation,” and (iii) populations that include “people living in severely inadequate and insecure accommodation.” The assessment revealed that “staying at home” was physically and practically unattainable across groups. The study identified specific institutional, social, and cultural challenges apart from the common economic crisis, whereas the utilization of social welfare was low. Findings suggest that the rapid increase of groups classified as “(i)” and “(ii)” should be addressed by engaging stakeholders to enhance the availability and accessibility of social welfare and rescue measures, and to ensure safe and private accommodations for all groups. It is critical to enhance multi-sectoral collaboration in responding to the common and specific vulnerabilities of these population groups from health, socio-economic, and humanitarian perspectives. Under the pandemic, homelessness should be regarded less as a peculiar problem for specific populations but an extension of daily life. The framework can be a reference when planning the comprehensive yet concise assessment of populations with unstable housing in other countries to inform responses to the pandemic.

## Introduction

Coronavirus disease 2019 (COVID-19) affected people at the global scale and brought a devastating impact on disadvantaged populations [1]. Assessing members of society likely to be affected by COVID-19 is essential to effectively respond to the pandemic in each country as entities classified under disadvantaged groups can change [2]. Especially, homeless individuals are affected by the COVID-19 pandemic [3].

Homeless people, who are reported to carry five- to tenfold mortality rates compared with other groups, are engaged in situations prone to COVID-19 infection [4, 5]. These individuals are at high risk of progressing to severe conditions due to underlying chronic diseases [6]. However, measures for preventing COVID-19 transmission, such as staying at home, rigorous hygiene, and strict social distancing, are reportedly unrealistic for the homeless [7].

From the viewpoint of Sustainable Development Goals (SDGs), homelessness is related to several issues, such as poverty, hunger, poor health, lack of access to education, clean water and sanitation, gender, inequalities, and barriers to achieving sustainable and inclusive cities [8]. Addressing homelessness during the pandemic requires a broad understanding of country-specific situations so that effective multi-stakeholder cooperation and solidarity can be explored through the lens of SDGs and their interlinked nature.

The definition of homelessness varies, which tends to be conceptualized not only by accommodation but also by broad perspectives, such as security of tenure, physical environment of dwelling, and social interaction, as proposed by the Institute of Global Homelessness (IGH) [9]. The IGH classified the homeless into “people without accommodation,” “people living in temporary or crisis accommodation,” and “people living in severely inadequate and/or insecure accommodation.” In Japan, the definition of homelessness is relatively narrow [10, 11] and people without accommodation are frequently highlighted. Many people living in temporary, inadequate, or insecure accommodation tend to be invisible to society [12, 13]. Groups with diverse characteristics, such as non-regular employment; lesbian, gay, bisexual, transgender, and queer or questioning (LGBTQ); youth; foreign-origin; and internal displacement, were reported among the latter populations, and COVID-19 may force such groups to face common and specific challenges. However, the condition of these populations remains undocumented or lacks comparative analysis.

Since we could not find any existing tool to conduct such analysis, we drafted a framework that consisted of two dimensions, namely (i) three categories of populations with unstable housing conditions based on the IGH classification, and (ii) COVID-19 related situation and response concerning respective populations, encompassing health, socio-economic, and humanitarian elements. Consideration was given to comprehensiveness and conciseness for developing the framework so that stakeholders of different populations could have a good overview for communication and collaboration across sectors.

Regarding the first dimension, we adjusted the third population of the IGH classification from “people living in severely inadequate and/or insecure accommodation” to populations that include “people living in severely inadequate and/or insecure accommodation.” This adjustment was made to include people who do not necessarily reside in “severely inadequate and/or insecure accommodation,” but are susceptible to losing accommodation, considering the potential devastating impact of COVID-19 pandemic on housing of a broad range of populations [14].

The second dimension involved characteristics of people including their vulnerabilities, socio-economic consequences of COVID-19, risk of COVID-19 infection and progression, existing and urgent measures taken, and remaining challenges, in line with the COVID-19 operational response strategies for the United Nations (UN) families and partners [15]. These strategies included COVID-19 Strategic Preparedness and Response Plan (SPRP) for the health sector [16], UN Socio-Economic Response Framework [17], and Global Humanitarian Response Plan (GHRP) [18]. Each of these strategies and related documents highlighted the importance of vulnerable populations including homeless people.

We presented the draft framework to experts and practitioners engaged in unstable housing conditions, including those with expertise and experiences in community health, mental health, and social determinants of health, as well as housing support, livelihood support, and social welfare. They provided suggestions and advice to refine categorization of populations and identify nine socially distinguishable groups of individuals, based on their experiences in working with NGOs, public sector, and academia.

We then applied the framework to assess populations facing difficulties in securing accommodation in Japan with a focus on Tokyo, who were reported to have a wide range of background and characteristics [19–21]. These nine groups were classified into the abovementioned three categories:

- “People without accommodation” refers to individuals living on streets or open spaces.
- “People living in temporary or crisis accommodation” comprises three groups, namely (i) free- or low-cost accommodations and public assistance facilities, (ii) self-reliance support centers, and (iii) cyber-homeless.
- Populations that include “people living in severely inadequate and/or insecure accommodation” encompass the remaining five groups, namely (i) non-regular workers and self-employed individuals including female-parent household in industries susceptible to repercussions from the COVID-19 pandemic; (ii) impoverished LGBTQ; (iii) evacuees from Fukushima after the nuclear disaster (internally displaced people in Japan); (iv) migrants—technical interns, foreign students, poverty-stricken long-term residents, and undocumented foreigners; and (v) adolescents and children abused in domestic settings.

For each category, a literature review was conducted in terms of characteristics and vulnerabilities, socio-economic consequences of COVID-19, risk of COVID-19 infection and progression, existing measures most relevant to the vulnerabilities, and urgent measures to address the socio-economic consequences of COVID-19 and risk of COVID-19 infection and progression. The literature included journal articles, reports, government documents, and news articles as of May 31, 2020 (The first COVID-19 case was reported on 16 January 2020 in Japan. The number of reported cases increased to 16,851 by 31 May 2020. The Japanese Government issued an emergency declaration on 7 April 2020, and it was extended to 25 May 2020). Tables were constructed and contents refined after consultation with experts for comparative analysis. The following sections present an overview of social security schemes in Japan as background information of the review, main findings of the review, and strategic directions to address challenges.

## Overview of social security schemes in Japan

Main social security schemes in Japan include public pension systems, health care systems, public assistance, labor insurance, social welfare for the elderly, family policies, and policies for persons with disabilities [22]. Income security for the elderly, disabled person, and survivors is provided by the public pension systems. Health care systems include public health, health insurance, and maternal and child health services. Public assistance is offered as part of the financial support system for the poor. Employment insurance, work-related accident insurance, and others are provided as part of the worker protection system. Social welfare for the elderly includes long-term care insurance. Family policies involve childcare and financial support such as child allowance and support for single-parent households. Policies for persons with disabilities include care service provision and financial assistance.

## People without accommodation and people living in temporary or crisis accommodation (Table 1)

### Characteristics and vulnerabilities of populations

i) People without accommodation are defined as those living their daily lives in city parks, riverside, streets, stations, and other facilities according to the Act on Special Measures concerning Assistance in Self-Support of Homeless. They are predominantly male and aged over 60 years [10, 23], with high prevalence of mental illnesses, intellectual disability, and chronic diseases, such as hypertension, diabetes, and alcoholism [19].
ii) People living in temporary or crisis accommodation include the following groups:
  - People in free- or low-cost accommodations and public assistance facilities are mostly beneficiaries of the Public Assistance System which aims to guarantee the minimum standard of living [29, 49]. Free- or low-cost accommodations offer accommodation only (with occasional food and consultation services), whereas public assistance facilities provide livelihood assistance and in most cases care for physical or mental disabilities. However, a high prevalence of mental illnesses and intellectual disability was reported among people in free- or low-cost accommodations. Furthermore, economic exploitation by owners of free- or low-cost accommodations and sub-optimal dwelling environment were highlighted [10].
  - Self-reliance support centers [50] are to provide temporary living assistance as stipulated by the Act on Self-reliance Support for Needy Persons, where people are required to find a job and stay for up to 6 months.
  - The cyber-homeless stay inside internet or comic book cafés, which are open for business 24 h a day and offer not only internet and/or comic book services, but also food, drink, showers, and private rooms. These facilities are often used as affordable temporary accommodation. According to the surveys conducted by the Tokyo Metropolitan Government in 2016, more than a quarter of overnight users were staying these cafés because they had lost homes. Among them, more than 80% were non-regular workers or self-employed individuals. The majority of users are male and lack employment and health insurance [33]. Some of them also sleep on the streets when needed.

**Table 1:** People without accommodation and people living in temporary or crisis accommodation

	People without accommodation	People living in temporary or crisis accommodation		
		Free- or low-cost accommodation and public assistance facilities	Self-reliance support centers	Internet café or comic book café
**Characteristics and vulnerabilities**	• A total of 4253 in Japan, and 1037 people in Tokyo alone, the majority of whom are males aged over 60 [10, 23]. • Night-time count of homeless people by NGO indicated an actual number that is 2.8 times higher than that produced by a public survey in Tokyo [24]. • High prevalence of mental illness, intellectual disability, and chronic diseases, such as hypertension, diabetes, and alcoholism [19, 23, 25]. • Threatened by natural disasters, eviction requests, and raids [26, 27].	• Free- or low-cost accommodations shelter 15,600 people in Japan [28] and 4069 people in Tokyo alone, out of which 3779 are recipients of public assistance [26]. • Public assistance facilities accommodate 35,918 people [29]. • Congregated dwelling environment [28]. • TB infection clusters reported [30].	• Utilized by a total of 16,641 people in Japan in 2019 [21]. • Capacity for accommodation is 350 people in Tokyo [31]. • Tenancy terminates in 6 months [31]. • Inmates are required to follow rules, such as alcohol ban and curfews, which drives them to escape [32].	• The number of people that utilize such cafes is 5000 for Japan (2007) and 5055 for Tokyo (2016). The majority are males aged between 30 and 59 years [33]. • Frequent visitors are mainly non-regular workers without employment or health insurance. Oftentimes, many sleep on the streets [34]. • Despite its availability, the existing social welfare is not utilized [34]. • TB infection clusters reported [34].
**Socio-economic consequences of COVID-19**	• Loss of income [35]. • Soup kitchen services are on hold [36]. • Increase in number of people on the streets [37].	• Those who are not welfare recipients lose job income [38].	• Difficulty of finding a job during the 6-month tenancy period [32].	• Loss of income [39]. • Suspension of internet cafés, which deprives accommodation [40].
**Risks of COVID-19 infections and progression**	• Aging and chronic diseases [23]. • Difficulty performing hand hygiene practices [37]. • Delay in access to medical services [23].	• Sharing a small room with several people [28].	• Sharing a room and amenities, no rooms are individually available, particularly in Tokyo [31].	• Sharing a big space and amenities [36]. • 61.2% of them are without health insurance [33]. • Delay in access to medical services [23].
**Existing measures most relevant to vulnerabilities**	• Public Assistance System for Guaranteeing Minimum Standard of Living [41]. • Self-reliance Support for Needy Persons [42].	• Public Assistance System for Guaranteeing Minimum Standard of Living [41].	• Self-reliance Support for Needy Persons [42].	• Self-reliance Support for Needy Persons [42]. • Tokyo Challenge Net [43].
**Urgent measures for COVID-19 consequences and risks**	• MHLW circular on streamlining approval procedures of public assistance [44]. • MHLW circular on single rooms for the newly needy [45].	• Infection control manuals [46].	• Infection control manuals [47].	• MHLW circular on streamlining approval procedures of public assistance [44]. • MHLW circular on single rooms for the newly needy [45].
**Remaining challenges for COVID-19 consequences and risks**	• Low utilization (20%) of the Public Assistance System due to tedious local government procedures and stigma against the system [48]. • Implement single room arrangement. • Access to medical services.	• Despite infection control practices, multiple inmates continue to share rooms [46].	• Despite infection control practices, multiple inmates continue to share rooms [47].	• Low utilization (20%) of the Public Assistance System due to tedious local government procedures and stigma against the system [48]. • Implement single room arrangement. • Access to medical services.

### Socio-economic consequences of COVID-19

Loss/reduction of income or difficulty in finding a job due to COVID-19 exerted negative consequences on all groups. People without accommodation gained reduced access to soup and kitchen services offered by NGOs. The suspension of internet cafés resulted in the increase of people without accommodation.

### Risk of COVID-19 transmission and progression

All groups classified as people living in temporary or crisis accommodation typically stay in shared spaces. People experiencing loss/reduction of income, particularly those without accommodation and cyber-homeless, are prone to losing health insurance, which increases the difficulty of accessing medical services including COVID-19 testing.

### Measures and remaining challenges

Although the Public Assistance System guarantees the minimum standard of living, the take-up rate of Public Assistance reached as low as 20% in Japan [51]. The contributing factors include social stigma against users, limited awareness of the system, cumbersome application procedures, and strict approval criteria [52]. Amid the COVID-19 pandemic, the Ministry of Health, Labour and Welfare (MHLW) issued circulars to local governments to streamline the application and approval procedures of the Public Assistance System and to arrange single rooms for individuals who recently lost accommodation. In addition, the government initiated Special Cash Payments amounting to JPY100,000 targeting residents under the Basic Resident Registration System.

The remaining challenges for addressing the socio-economic consequences of COVID-19 and risk of COVID-19 infection and progression include (i) the low utilization of the Public Assistance System and social stigma against users [52]; (ii) effective and efficient implementation of single room arrangements for individuals who lost accommodation; (iii) prevention practices and/or single room arrangements and other measures for people living in self-reliance support centers, free- or low-cost accommodations, and public assistance facilities; and (iv) access to medical services, particularly among people not covered by health insurance.

## Populations that include people living in severely inadequate and insecure accommodation (Table 2)

### Characteristics and vulnerabilities of populations

i) Non-regular workers and self-employed individuals including female-parent households include populations with housing instability. The wage for non-regular workers was 65% of that of regular workers in 2018 [101], whereas the relative poverty rate of single-parent families (mostly female-parents) was 50.8%. Furthermore, non-regular workers are susceptible to termination of employment contract during economic recessions [102]. The perils of workers are exacerbated by the nearly halved reduction of income [103] due to the low-level unemployment benefit of employment insurance, particularly for individuals with short work tenure [104].
ii) The LGBTQ appear to include significant segments of people facing housing instability. A web-based survey that targeted men who have sex with men (MSM) in 2016 revealed that 5.2% out of 6921 respondents had ever lost accommodation, whereas 56.1% experienced worry or stress over income or debt [59]. Moreover, a survey targeting property owners in 2018 indicated that less than 40% welcome same-sex couples as tenants [60].
iii) Evacuees from Fukushima after the nuclear disaster in 2011 (internally displaced people) experienced job losses in their home town and were forced to find new jobs as non-regular employees [105]. Families are separated physically, which results in increased household expenses [106]. In addition, these workers lost social capital, which is difficult to rebuild in the destination community. Discontinuation of housing assistance as stipulated in the Disaster Relief Act of 2017 affected more than 12,000 households.
iv) Migrants—technical interns (which are foreign nationals in Japan under the Technical Intern Training Program of the Japanese Government), international students, asylum seekers, poverty-stricken long-term residents, and undocumented foreigners have various vulnerabilities. Specifically, technical interns face labor, health, and safety issues; are forced to stay in sub-optimal housing; and are burdened with huge debts in their home countries [107]. In fact, more than 9000 technical interns have disappeared from designated work places in 2019. International students tend to depend on heavy part-time jobs and have huge debts in their home countries [108]. Long-term residents, especially those of Japanese descent, work in unstable employment conditions [109]. Asylum seekers are banned from working for 6 months, and many are detained in immigration detention centers [110]. Undocumented foreigners, such as ex-technical interns and ex-international students, are excluded from health insurance or social protection schemes [111].
v) A large number of adolescents and children roam the streets due to physical and/or mental abuse in their domestic settings [112]. A total of 230,000 roaming and 160,000 abuse cases were reported in 2018, which have been rapidly increasing in the past decade [73, 113]. The prevalent type of abuse was psychological, followed by physical, neglect, and sexual abuse. Abused adolescent girls roaming the streets face various challenges, such as sexual exploitation [114].

**Table 2:** Populations that include people living in severely inadequate and insecure accommodation

	Non-regular workers and self-employed individuals including single-female-parent household	Impoverished LGBTQ	Internally displaced people (evacuees from Fukushima after nuclear disaster)	Migrants—technical interns, international students, undocumented migrants, long-term foreign residents, and undocumented foreigners	Adolescents and children abused in domestic settings
**Characteristics and vulnerabilities**	• 2.6 million out of 21 million non-regular workers reluctantly took non-regular jobs due to lack of regular employment opportunities [53]. • Non-regular workers’ wage is 65% of regular workers’ wage [54]. • In 1.2 million single-female-parent household, 47% are non-regular workers or self-employed [55]. • The relative poverty rate of single-parent families is 50.8% [56]. Income of single-female-parent household is 38% of that of household with children [57].	• LGBTQ accounts for 10% of the population aged between 20 and 60 years [58]. • A total of 5.2% of MSM web-survey respondents ever lost accommodation, 22.7% sold sex, and 56.1% worried or stressed about income or debt [59]. • Less than 40% of property owners welcome same-sex couples as tenants [60]. • Sexual minorities may face discrimination in terms of access to housing [61].	• 30,000 people are not allowed to live in their home town in Fukushima if the area is classified as a mandatory evacuate zone as of April 2020 [62]. • A sizeable number of people voluntarily evacuated from their residential areas [63]. This number was estimated at 31,000 as of 2015 [64]. • After evacuation, non-regular workers among Fukushima evacuees increased [64], income decreased, and expenditure increased [64]. • Housing support based on the Disaster Relief Act ended in 2017, which affected more than 12,000 households [65]. • Blending into destination communities is difficult partly due to stigma and discrimination against Fukushima evacuees [66].	• A total of 410,972 technical interns were noted as of 2019, out of which 2.1% (9,052 people) are reported to have disappeared and may be labeled as undocumented foreigners [67]. • 83,811 foreign students are studying at language schools [68], many of whom are considered cheap labor [69]. • Out of 10,000 asylum seekers per year in Japan, only 40 are accepted [70]. Immigration detention centers reported 1253 detainees as of June 2019 [71]. • 2,829,416 long-term residents, including Japanese descents [72]. • Undocumented foreigners are excluded from social protection schemes.	• 230,000 adolescents and children roam the streets at night [73]. • Physical and/or mental abuse in a domestic environment drive them away from home and out on the streets [74]. • In 2018, 160,000 adolescent and child abuse cases (up to 18 years old) were reported [75]. • The most prevalent type of abuse was psychological abuse, followed by physical abuse, neglect, and sexual abuse [75, 76]. • Sexual exploitation of adolescent girls who roam the streets [77].
**Socio-economic consequences of COVID-19**	• Loss of income [78]. • Increased food expenses due to school closure [79].	• Threatened by enforcement of revealing sexual orientation in the case of contracting the novel coronavirus [80].	• Loss of income [78]. • Suspension of regular exchange events by evacuees [81].	• Loss of income [82]. • Loss of resident status (Visa expiration) [83].	• Worsened cases of child/adolescent abuse [84]. • Less opportunities for recognizing child/adolescent abuse due to school closure [85].
**Risk of COVID-19 infections and progression**	• Only 17.0% of non-regular workers are working from home compared with 27.9% of regular workers [86]. • Delay in access to medical services due to lack of health insurance [87].	• Inevitability of going out for work. • Delay in access to medical services due to lack of health insurance [87].	• Inevitability of going out for work. • Delay in access to medical services due to lack of health insurance [87].	• Inevitability of going out for work. • Delay in access to medical services due to lack of health insurance [87]. • Sharing a room [88].	• Driven to flee from child/adolescent abuse and gather in closed spaces [89]. • Sharing a room and amenities at a temporary custody facility [90].
**Existing measures most relevant to vulnerabilities**	• Unemployment benefit of employment insurance [91]. • Job applicant benefits for daily workers [92].	• Unemployment benefit of employment insurance [91]. • Job applicant benefits for daily workers [92].	• Unemployment benefit of employment insurance [91]. • Job applicant benefits for daily workers [92].	• Unemployment benefit of employment insurance [91]. • Job applicant benefits for daily workers [92].	• Act on the Partial Amendment to the Child Welfare Act and other legal measures to enhance the efforts for preventing child abuse [93].
**Urgent measures for COVID-19 consequences and risk**	• Employment adjustment subsidy [94]. • Subsidy Program for Sustaining Businesses Starts [95].	• Employment adjustment subsidy [94]. • Subsidy Program for Sustaining Businesses Starts [95].	• Employment adjustment subsidy [94]. • Subsidy Program for Sustaining Businesses Starts [95].	• Employment adjustment subsidy [94]. • Flexibility in changing Visa status [96].	• Action plan to strengthen child abuse monitoring [97].
**Remaining challenges to address COVID-19 consequences and risks**	• Supporting individuals not covered by any measures [98]. • Reducing barriers to the application of existing and urgent measures [99]. • Preventing transmission during work [100].	• Supporting individuals not covered by any measures [98]. • Reducing barriers to the application of existing and urgent measures [99]. • Preventing transmission during work [100].	• Supporting individuals not covered by any measures [98]. • Reducing barriers to the application of existing and urgent measures [99]. • Preventing transmission during work [100]. • Mitigating the isolation of evacuees, particularly the elderly [81].	• Supporting individuals not covered by any measures [98]. • Reducing barriers to the application of existing and urgent measures [99]. • Preventing transmission during work [100]. • Understanding the situation and responding to the needs of undocumented foreigners	• Enhancing cooperation among schools, local governments, and child guidance centers during school closure [85]. • Developing alternative approaches to activities of existing public institutions to meet the needs of children and adolescents [77].

### Socio-economic consequences of COVID-19

Loss/reduction of income exerted negative consequences on non-regular workers, impoverished LGBTQ, internally displaced people from Fukushima, and foreigners, particularly in industries susceptible to repercussions from the pandemic. Furthermore, each population faces additional difficulties. For example, admission to a facility (i.e., free- or low-cost accommodation) where single rooms are unavailable is traumatic for the LGBTQ, and unemployment of foreign workers leads to loss of residence status and access to social and health services. Finally, staying indoors results in increases in adolescent and child abuse cases [115].

### Risk of COVID-19 transmission and progression

Despite the stay-at-home policy, people belonging to the five groups are forced to go out for work to earn a living or flee from domestic abuse. People with loss/reduction of income are prone to losing health insurance as they experience difficulties in paying the insurance premium, which may result in delay in accessing medical services including COVID-19 testing. People detained in immigration centers, children and youth in the Child Guidance Center’s Temporary Care Home, and a portion of technical interns are staying in congregated settings. The housing conditions of asylum seekers and undocumented foreigners including ex-technical interns and ex-students remain unclear.

### Measures and remaining challenges

The eligibility criteria for the unemployment benefits of employment insurance have been expanded over time [73]. However, people who lost jobs, particularly non-regular workers ineligible for unemployment benefits, are prone to losing health insurance. At least 180,000 households are required to pay the full amount of medical cost when using medical services due to delinquency in payment [116].

As part of urgent measures to respond to the pandemic, the government established the Employment Adjustment Subsidy for employers and the Subsidy Program for Sustaining Businesses for self-employed and freelance workers [94, 117]. The former is “provided for an employer, who has been forced to reduce business activities due to the effects of COVID-19 and temporarily suspended work, or who has trained or dispatched employees in order to maintain their employment, for some of the leave allowance paid to employees,” and the latter “targets companies facing severe conditions in particular and provides them with subsidies for a wide variety of purposes that in general are considered effective in supporting them in sustaining or reviving their businesses.” Furthermore, the government initiated Special Cash Payments amounting to JPY100,000 targeting the country’s population registered under the Basic Resident Registration System as eligible recipients.

The remaining challenges common to all groups except for people in abusive domestic settings include providing support for individuals not covered by existing and urgent measures and reducing barriers to such measures. Particularly, assessing the situation of undocumented foreigners is imperative to the development of adequate responses [111].

For adolescents and children abused in domestic settings, enhancing cooperation among schools, local governments, and child guidance centers during school closure due to COVID-19 is crucial [118]. To meet the diverse needs of adolescents, such as protection from sexual exploitation and isolation, alternative approaches to existing public institutions should be developed and expanded, which include outreach programs, peer support, and adolescent-sensitive services [114].

## Strategic directions to address challenges

The framework used for the assessment integrated the IGH definition of homelessness and the health, socio-economic, and humanitarian perspectives in line with the COVID-19 operational response strategies for the United Nations (UN) families and partners. While the scope of the assessment could be deemed ambitious, this assessment illustrated a comprehensive yet concise picture of nine groups of populations with unstable housing conditions with diverse backgrounds in Japan.

These populations share a common problem. “Staying at home” is unrealistic because they lack adequate homes as a fundamental human right or are compelled to venture outside to earn a living. Furthermore, the assessment identified a range of major challenges as indicated in Tables 1 and 2, to wit: increasing utilization of the Public Assistance System, effective and efficient implementation of urgent measures, prevention practices in congregated settings, preventing transmission during work outside home, ensuring timely access to medical services (particularly among individuals not covered by health insurance or other schemes), understanding and addressing the situation of undocumented foreigners, and protecting children and adolescents abused in domestic settings.

To address these challenges, which is a huge task for society, the findings suggest and inspire the following strategies:

- Economic damages caused by the pandemic force a large number of populations indicated in Table 2 to confront existing problems faced by the populations summarized in Table 1. The existing problems include low utilization of the Public Assistance System, and sub-optimal conditions of many self-reliance support centers and free- or low-cost accommodations. With a view to converting crisis into opportunity, it is crucial to highlight and address the existing problems from the viewpoint of people with diverse backgrounds in need of the system and centers due to COVID-19. Specifically, efforts are necessary to engage stakeholders to enhance the implementation and utilization of the Public Assistance System and advocate a safe and private accommodation for all groups.
- All groups were deemed to feature vulnerabilities that require vigilant consideration in designing assistance schemes and operationalizing procedures. These vulnerabilities surfaced by the COVID-19 pandemic include traumatic life experiences, mental illnesses, intellectual disability, substance use, gender-related issues, lack of social capital, unstable employment, financial debt, migration, language barriers, and domestic violence.
  ▪ Each group is not mutually exclusive and may overlap one another. Thus, efforts to address vulnerabilities should be maximized across initiatives that support different groups. For instance, a consortium of NGOs was established in 2019 to gather and pool funding for emergency support for individuals who lack accommodation [12]. The consortium consisted of NGOs working on various population groups, such as people on streets and in temporary accommodations, adolescent girls in abusive domestic environments, women with pregnancy-related issues, asylum seekers and refugees, poor children and parents, youth in poverty, internally displaced people, and victims of human trafficking, as well as housing support, research and advocacy, and labor issues.
  ▪ It is also critical to enhance multi-sectoral collaboration in responding to the vulnerabilities of respective groups from health, socio-economic, and humanitarian perspectives [14]. In particular, the health sector is expected to play an active role to foster such collaboration in addressing the needs of these groups through working with social welfare, labor, industry, education, and other sectors. Initiatives targeting these groups should be advocated and mainstreamed, building on existing initiatives such as Inclusion Health, which is a service, research, and policy agenda that aims to prevent and redress health and social inequities among the most vulnerable and excluded populations such as homeless, prisoners, and drug users [119, 120].

## Conclusion

Viewing the spectrum of population groups with unstable housing conditions using the proposed framework that encompasses health, socio-economic, and humanitarian perspectives, the COVID-19 pandemic seemingly teaches a lesson, that is, homelessness is not a problem for a specific population but an extension of daily life that many citizens can face with a subtle trigger. A variety of issues that emerged regarding homelessness provided opportunities to recognize and address old and new social determinants of health and to move toward a sustainable symbiotic society in synergy with initiatives for tackling other social issues toward SDGs.

It should be noted that the framework was developed and applied to a wide range of population groups in Japan through consultation with experts and practitioners engaged in unstable housing conditions. However, given that the possibility of overlooking other elusive populations cannot be ruled out, further studies are needed to refine this framework. Considering elusiveness and changeability of vulnerable populations, we hope that this framework can be a reference when planning the comprehensive yet concise assessment of populations with unstable housing in other countries [2]. The authors wish to share their insights so that partnership and solidarity could be forged across countries [121].

## Limitations

Instead of a systematic manner, data collection was performed according to the framework developed. Thus, its validity and generalizability should be further examined. Although not discussed, other populations exist in Japan, i.e., individuals who are not homeless but significantly susceptible to COVID-19. Such persons include those with disabilities in facilities, the elderly in nursing homes, children in orphanages, prisoners, and psychiatric patients with prolonged hospital stay.

## Data Availability

Not applicable, because this article reviews existing literature.

## Abbreviations

COVID-19: Coronavirus disease 2019
GHRP: Global Humanitarian Response Plan
IGH: The Institute of Global Homelessness
LGBTQ: Lesbian, gay, bisexual, transgender, and queer or questioning
MHLW: Ministry of Health, Labour and Welfare
MSM: Men who have sex with men
SDGs: Sustainable Development Goals
SPRP: Strategic Preparedness and Response Plan
UN: United Nations
